# Buffered formic acid and a monoglyceride blend improve performance and modulate gut bacteria and immunity gene expression in broilers under necrotic enteritis challenge

**DOI:** 10.1016/j.psj.2023.102978

**Published:** 2023-08-02

**Authors:** Ali Daneshmand, Nishchal K. Sharma, Sarbast K. Kheravii, Leon Hall, Shu-Biao Wu

**Affiliations:** ⁎ School of Environmental and Rural Science, University of New England, Armidale, NSW 2351 Australia; † BASF Australia Ltd, 12/28 Freshwater Place, Southbank, VIC 3006, Australia

**Keywords:** broiler chicken, buffered formic acid, necrotic enteritis, immunity, gut health

## Abstract

Due to the removal of antibiotics from animal feed, alternatives have been sought to control necrotic enteritis (**NE**) in broilers. The current study investigated the effects of buffered formic acid (Amasil NA) and monoglycerides of short- and medium-chain fatty acids (Balangut LS P) on the performance and gut health of broilers challenged with NE. A total of 816 as-hatched 1-d-old chicks (Cobb 500) were randomly assigned to 6 treatments with 8 replicates. Treatments were: T1) nonchallenged control; T2) NE challenged control; T3) Amasil NA (challenge plus Amasil NA, 0.3% throughout all phases); T4) Balangut LS P (challenge plus Balangut LS P, 0.5%, 0.3%, and 0.2% in the starter, grower and finisher phases, respectively; T5) Combined (challenge plus combination of T3 and T4); T6) Antibiotic (challenge plus Zn bacitracin, 0.05 % throughout all phases). Birds were orally gavaged with live *Eimeria* vaccine species (d 9) and with *Clostridium perfringens* (d 14 and 15). On d 16, birds were sampled to evaluate gut permeability, microbiota, and mRNA abundance in the jejunum. The data were analyzed in JMP software using one-way ANOVA with Tukey's test to separate means, and Kruskal-Wallis test was used for non-normally-distributed parameters. Results showed that Balangut LS P decreased (P<0.05) feed conversion ratio compared to nonchallenged ones at the end of the study. Balangut LS P reduced (*P* < 0.05) the level of cecal *Bacteriods* compared to nonchallenged group, whereas Amasil NA shifted the levels of ileal *Bifidobacteria*, Enterobacteriaceae, and *Lactobacillus* towards nonchallenged control (*P* > 0.05). NE challenge upregulated (*P* < 0.001) the expression of IL-21R, zeta chain of T cell receptor (ZAP70), and dual specificity phosphatase 4 (DUSP4) compared to nonchallenged birds, whereas Balangut LS P showed an intermediate (*P* > 0.05) expression pattern of these genes towards nonchallenged and antibiotic groups. In conclusion, combination of Balangut LS P and Amasil NA has the potential to be used as an additive to improve the performance and gut health of broiler chickens, especially under challenging conditions such as NE infections.

## INTRODUCTION

Necrotic enteritis (**NE**) is an enteric poultry disease that decreases production, increases mortality, compromises the welfare of the animals, and increases veterinary costs (Wade and Keyburn, 2015). *Clostridium perfringens* is the known cause of NE that inhabits the intestine of poultry in relatively low numbers (<10^4^ cfu) (Asaoka et al., 2004). It has been reported that the presence of predisposing factors, such as pathogens (e.g., Eimeria) and diet ingredients (e.g., fish meal), can increase the number of NetB-positive *C. perfringens* (>10^7^ cfu) in the intestine leading to NE in broilers. It has been estimated that annual global costs for the poultry industry can be up to US$6 billion (Wade and Keyburn, 2015). Although the use of in-feed antibiotics was the common strategy to control NE in poultry, its use was banned in the European Union or is voluntarily phasing out worldwide due to the emergence of antibiotic-resistant bacteria, which poses a health risk for humans (Aarestrup et al., 2008). This has led to the search for alternatives to antibiotics, resulting in the introduction of various additives to the poultry industry, such as probiotics, prebiotics, phytogenics, and organic acids.

Organic acids are known for their antibacterial effects on the intestine of poultry (van Immerseel et al., 2006). The antibacterial effects of organic acids have been attributed to the change of pH change in the gut, disrupted bacterial layers, and consequent interference of the intracellular metabolisms of bacteria (van Immerseel et al., 2006). The beneficial effects of organic acids on performance, immunity, nutrient digestibility, buffering capacity, and antibacterial activity against *Salmonella* and *Escherichia coli* have been reported in poultry (van Immerseel et al., 2006; Hajati, 2018). Furthermore, while formic acid showed no significant effects on performance, intestinal morphology, and plasma metabolites (Hernández et al., 2006), and is corrosive to the gastrointestinal tract, the buffered form improved performance, immune parameters, and gut health in broilers (Ragaa and Korany, 2016). More recently, 2 studies were conducted to evaluate the efficiency of a blend of monoglycerides of short and medium-chain fatty acids (SMCFA, Balangut LS P) and buffered formic acid (Amasil NA) in broilers under NE challenge. In the first study, Gharib-Naseri et al. (2021b) examined high and low levels of these products and demonstrated that both levels of Balangut LS P significantly improved feed conversion ratio (**FCR**) during 0 to 35 d compared to the nonchallenged group, whereas low levels of Balangut LS P and Amasil NA improved FCR in the finisher phase, similar to the antibiotic group. In the second study, Kumar et al. (2021) supplemented Balangut LS P only during the starter phase with or without Amasil NA during the grower and finisher phases. They showed that Balangut LS P caused less lesion score, upregulation of tight junctional protein (**TJP1**), and higher gross energy digestibility compared to NE-challenged control group. Eventually, both studies speculated that the supplementation of these products during whole experimental phases and their combinations might beneficially affect broiler performance and gut health under the NE challenge. Therefore, this study aimed to investigate the potential of Amasil NA and Balangut LS P alone or their combination on performance, gut permeability, intestinal microbiota and mRNA abundance of broilers under NE challenge. The hypothesis was that a single or combined use of these products could modulate the intestinal microenvironment to enhance gut health, alter the intestinal bacterial population, and consequently alleviate the negative effect of NE challenge on the performance and health of the broilers.

## MATERIALS AND METHODS

The animal ethics committee of the University of New England approved the experimental protocols of this research (AEC 19-034).

### Birds and Housing

A total of 816 day-old as-hatched chicks (Cobb 500) were provided from a local hatchery (Baiada Pty Ltd, Tamworth, NSW, Australia). Upon arrival, chicks were weighed randomly and divided into 6 treatments, consisting of 8 pens with 17 birds in each pen. The sex of birds in each pen (at least 8 birds) were determined on d 6 using high-resolution melting analysis with DNA extracted from feathers (England et al., 2021) to use for sampling with marked sexes. Pens (87 cm × 118 cm) including both nonchallenged and challenged groups were placed in an environmentally controlled room, filled with wood shavings (5cm), and equipped with tube feeders and nipple drinkers. The guidelines of Cobb-Vantress (2018a) were used to adjust the room lighting program, temperature, and ventilation. Feed and water were provided ad-libitum for the period of the experiment (d 35).

### Treatments and Diets

Experimental diets and the level of each additive at each phase were as follows: T1) Nonchallenged control; T2) Challenged control; T3) Amasil NA: challenged control with 0.30% Amasil NA (BASF SE, Ludwigshafen, Germany) supplemented in feed in all periods; T4) Balangut LS P: challenged control with 0.50%, 0.30%, 0.20% Balangut LS P (BASF SE, Ludwigshafen, Germany) supplemented in starter, grower, and finisher, respectively); T5) Combined: challenged control with the supplementation of a combination of Amasil NA and Balangut LS P (combination of T3 and T4); T6) antibiotic; challenged control with antibiotic (Zn-Bacitracin, 0.05% throughout all the phases) supplemented in feed.

Crude protein, amino acids, crude fiber, and crude fat of each feed ingredient were analyzed using NIRS (Evonik AminoProx, Frankfurt, Germany) before diet formulation. Experimental diets were based on wheat, soybean meal, and sorghum (Table 1), formulated to meet or exceed the minimum nutrient requirements of Cobb500 (Cobb-Vantress, 2018a, 2018b). All diets were passed through a pellet press (Palmer Milling Engineers Pty Ltd, Griffith, NSW, Australia) to produce a crumble diet for the starter phase. The same pellet press with different dies was used to pellet diets for the grower and finisher phases.

**Table 1:** Composition of experimental diets.^1^

Ingredients (%)	Starter (d 0–9)	Grower (d 10–21)	Finisher (d 22–35)
Wheat	52.2	53.7	57.9
Sorghum	10.0	15.0	14.7
Soybean meal	28.7	22.8	18.7
Meat meal	4.1	3.5	2.7
Canola oil	3.0	2.9	3.9
Limestone	0.759	0.765	0.769
Salt	0.192	0.176	0.166
Sodium bicarbonate	0.250	0.250	0.250
Choline chloride (75%)	0.059	0.078	0.068
L-lysine HCl	0.265	0.323	0.329
DL-Methionine	0.284	0.271	0.251
L-threonine	0.108	0.085	0.072
Vitamin premix^2^	0.075	0.075	0.075
Mineral premix^3^	0.100	0.100	0.100
Enzyme^4^	0.010	0.010	0.010
Total	100.0	100.0	100.0
Calculated nutrients			
AMEn, kcal/kg	3,014	3,062	3,162
Crude protein, %	25.0	22.7	20.9
Crude fiber, %	2.8	2.6	2.5
Ether extract, %	5.3	5.2	6.2
Dig. Lysine, %	1.220	1.120	1.020
Dig. Methionine, %	0.582	0.544	0.505
Dig. Met + Cys, %	0.910	0.850	0.800
Dig. Arginine, %	1.373	1.197	1.070
Dig. Threonine, %	0.830	0.730	0.660
Calcium, %	0.90	0.84	0.76
Phosphorus, %	0.68	0.64	0.60
Nonphytate P, %	0.45	0.42	0.38
Sodium, %	0.18	0.17	0.16
Chloride, %	0.24	0.24	0.23
Linoleic acid, %	1.20	1.18	1.40
Choline, mg/kg	1,700	1,690	1,550

1 Amasil NA (0.30% in all phases) and Balangut LS P (0.50%, 0.30%, 0.20% in starter, grower, and finisher, respectively) were supplemented to the diets.

2 Vitamin premix per kg diet: vitamin A, 12 MIU; vitamin D, 5 MIU; vitamin E, 75 mg; vitamin K, 3 mg; nicotinic acid, 55 mg; pantothenic acid, 13 mg; folic acid, 2 mg; riboflavin, 8 mg; cyanocobalamin, 0.016 mg; biotin, 0.25 mg; pyridoxine, 5 mg; thiamine, 3 mg; antioxidant, 50 mg.

3 Trace mineral concentrate supplied per kilogram of diet: Cu (sulfate), 16 mg; Fe (sulfate), 40 mg; I (iodide), 1.25 mg; Se (selenate), 0.3 mg; Mn (sulfate and oxide), 120 mg; Zn (sulfate and oxide), 100 mg; cereal-based carrier, 128 mg; mineral oil, 3.75 mg.

4 Natuphos E 5000 Combi G broiler 500 DCP.

### Necrotic Enteritis Challenge

The established method of inducing NE at the University of New England was applied with *Eimeria* and *C. perfringens* (Rodgers et al., 2015). On d 9, 1 mL live vaccine strains containing *Eimeria acervulina* (5,000 oocysts), *Eimeria maxima* (5,000 oocysts), and *Eimeria brunetti* (2,500 oocysts) (Eimeria Pty Ltd., Ringwood, VIC, Australia) was gavaged in all challenged chickens, whereas nonchallenged birds inoculated with sterile phosphate buffer solution. Challenged chickens were inoculated on d 14 and 15 with 1 mL culture of approximately 10^8^ cfu/mL *C. perfringens* (EHE-NE18, CSIRO, Livestock Industries, Geelong, Australia), whereas nonchallenged birds were gavaged with 1 mL of sterile thioglycolate broth, as a suspension of the bacterial culture.

### Bird Performance

Performance parameters, including average daily gain, average feed intake, and FCR, were calculated based on all chickens' weights and feed residuals of each pen at the end of starter (d 9), grower (d 21), and finisher (d 35). To adjust average feed intake and FCR, the number, weight, and sex of dead birds in each pen were recorded daily. At the end of the study, all birds were dissected to determine the sex of broilers in each pen, and sex ratio was used as a covariate for performance parameters in each phase.

### Sampling

On d 16, 2 males and 2 females from each pen were electrically stunned, blood samples were collected via jugular vein, and sacrificed to collect samples. The intestine was carefully detached from the carcass and divided into duodenum, jejunum, and ileum. The ileal and cecal sections were used to measure pH. The ileal and cecal contents were gently collected into a sterile tube, snap-frozen in liquid nitrogen, and stored at −20°C for subsequent DNA extraction for microbiota analysis. The intestinal sections were checked to score NE lesions based on a scale of 0 (none) to 6 (longest patches), as described previously (Keyburn et al., 2006). Two sections of approximately 2 cm were separated from the proximal jejunum of male broilers, washed in cold phosphate-buffered saline (**PBS**), immediately put in 2 mL safe-lock Eppendorf tubes containing RNAlater, kept in the fridge for 4 h, and finally preserved at −20°C for subsequent RNA extraction.

### Gut Permeability With Fluorescein Isothiocyanate Dextran

The method described by Vicuña et al. (2015) was used with some modifications to determine gut permeability with fluorescein isothiocyanate dextran (**FITC-d**). Briefly, 2 male and 2 female chickens from each pen were weighed, feather-marked, and orally gavaged with 1 mL autoclaved deionized water containing 4.17 mg FITC-d/kg BW (average molecular weight of 4,000, Sigma-Aldrich, Stockholm, Sweden) on d 16 about 2 h before sampling. The blood samples were collected in vacutainer tubes from the jugular vein by decapitation method, and centrifuged at 3,000 × *g* for 15 min to obtain serum samples. The sera were diluted (1:1 v/v) with PBS for further analysis. Approximately 40mg FITC-d were weighed and dissolved in 10 mL deionized water to provide the concentration of 4mg/mL which was then diluted with different amounts of PBS to produce a standard curve using different concentrations of FITC-d (4, 2, 1, 0.5, 0.25, 0.125, and 0.0625µg/mL). Then, the fluorescent levels in serum samples were recorded in a microplate reader (SpectraMax M2e, Molecular Devices, San Jose, CA) under an excitation wavelength of 485 nm and an emission wavelength of 528 nm. The concentrations of FITC-d in the serum samples were calculated and expressed as μg/mL serum.

### Ileal and Cecal pH

The pH of digesta samples from the ileum and cecum was measured by inserting a digital pH meter (Mettler-Toledo, UK) with a spear-tip pH electrode (Sensorex, Garden Grove, CA). Duplicate samples were measured for each bird, and the average value was used for statistical analysis.

### Bacterial DNA Extraction and Quantification

The QIAamp DNA Stool mini kit and the QIAamp 96 PowerFecal QIAcube HT Kit (QIAGEN GmbH, Hilden, Germany) were used to extract DNA from ileal and cecal contents, respectively, based on the manufacturer's instructions with slight modifications. Briefly, about 300 mg glass beads (0.1 mm) were placed in a 2 mL Eppendorf microtube, and approximately 190 and 100 mg ileal and cecal samples, respectively, were added to the tube containing glass beads. Then, different amounts of lysis buffer were added to each tube of ileal (i.e,. 400 µL ASL) and cecal (i.e., 500 µL prewarmed PW1) samples and thoroughly mixed by vortexing. The samples were homogenized using a TissueLyser II (QIAGEN GmbH, Hilden, Germany) at a frequency of 30/s for 5 min prior to being heated at 90°C for 15 min. The supernatant was transferred to new 1.5 Eppendorf tube after centrifugation. Then, the extraction procedures were followed based on the manufacturer's instructions. Ileal DNA was extracted manually following the method described by Kheravii et al. (2018) and cecal DNA was extracted using QIAcube HT automated system (QIAGEN GmbH, Hilden, Germany). The quantity and purity of extracted DNA samples were assessed on a NanoDrop ND-8000 spectrophotometer (Thermo Fisher Scientific, Waltham, MA). High-purity DNA with ratios of 260/280 and 260/230 greater than 1.8 was diluted 20 times with nuclease-free water and preserved at −20°C.

For PCR reactions, a SYBRGreen (SensiMix SYBR No-Rox, meridian Bioscience, Sydney, Australia) was applied for *Bacillus, Bacteroids, Bifidobacteria*, Enterobacteriaceae, *Lactobacillus, Ruminococcus,* and total bacteria using an qPCR machine (Rotor-Gene Q, QIAGEN GmbH, Hilden, Germany). The SensiFAST Probe No-ROX (Bioline, Sydney, Australia) was used to quantify *C. perfringens.* Table 2 shows the primers used for bacterial quantification expressed as log_10_ DNA copy number per gram of digesta.

**Table 2:** Sequences of primer pairs used for qPCR analysis of listed bacteria in ileal and cecal digesta.

Bacteria	Sequence (5′→ 3′)	Ta^°C^	Product size (bp)	References
Bacillus spp.	F-GCA ACG AGC GCA ACC CTTGA	63	92	Han et al., 2012
	R-TCA TCC CCA CCT TCC TCC GGT			
*Bacteroides* spp.	F-GAG AGG AAG GTC CCC CAC	63	106	Layton et al., 2006
	R-CGC TAC TTG GCT GGT TCA G			
*Bifidobacterium* spp*.*	F-GCG TCC GCT GTG GGC	63	106	Requena et al., 2002
	R-CTT CTC CGG CAT GGT GTT G			
*Lactobacillus* spp*.*	F-CAC CGC TAC ACA TGG AG	63	186	Fu et al., 2006
	R-AGC AGT AGG GAA TCT TCC A			
*Ruminococcus* spp*.*	F-GGC GGC YTR CTG GGC TTT	63	157	Ramirez-Farias et al., 2008
	R-CCA GGT GGA TWA CTT ATT GTG TTA A			
*C. perfringens*	F- CGCATAACGTTGAAAGATGG	58	105	Wise and Siragusa, 2005
	R- CCTTGGTAGGCCGTTACCC Probe-5′-FAM-TCA TCA TTC AAC CAA AGG AGC AAT CC-TAMRA-3			
Enterobacteriaceae	F- CAT TGA CGT TAC CCG CAG AAG AAG C	190	63	Bartosch et al., 2004
	R- CTC TAC GAG ACT CAA GCT TGC			
Total bacteria	F-CGG YCC AGA CTC CTA CGG G	63	204	Lee et al., 1996
	R-TTA CCG CGG CTG CTG GCA C			

### Jejunal mRNA Abundance

The Bioline ISOLATE II RNA Mini kit (meridian Bioscience, Sydney, NSW, Australia) was used to extract RNA from jejunal samples following the manufacturer's instructions. The extracted RNA was assessed for quantity and purity with NanoDrop ND-8000 spectrophotometer (Thermo Fisher Scientific, Waltham, MA) and for integrity with the Agilent 2100 Bioanalyzer (Agilent Technologies, Inc., Waldron, Germany). The RNA samples with a ratio of 260/230 greater than 2.0, 260/280 between 2.0 to 2.2, and an RIN of more than 7 were considered high quality. The SensiFast cDNA synthesis kit (meridian Bioscience, Sydney, NSW, Australia) was used to reverse-transcribe the extracted RNA into cDNA using the real-time PCR machine (Rotor-Gene 6000, Corbett, Sydney, NSW, Australia). The obtained cDNA was diluted 10 times with nuclease-free water and stored at −20°C.

The primers of target genes relating to gut integrity, transporters, and immunity are listed and specified in Table 3. qPCR was performed in duplicates using an SYBR Green kit (SensiFAST SYBR No-ROX, meridian Bioscience, Sydney, NSW, Australia) with a real-time PCR machine (Rotor-Gene Q, QIAGEN GmbH, Hilden, Germany). Different housekeeping genes were optimized for the most stable ones in response to the treatments of the current study using the geNorm module of qbase+ software (version 3.0, Biogazelle, Zwijnbeke, Belgium). These genes were: *Ribosomal protein L4* (**RPL4**)*, β-actin, glyceraldehyde 3-phosphate dehydrogenase* (**GAPDH**)*, hypoxanthine-guanine phosphoribosyltransferase* (**HPRT**)*, hydroxymethylbilane synthase* (**HMBS**)*, TATA box-binding protein (****TBP****), tyrosine 3-monooxygenase/tryptophan 5-monooxygenase* (**YWHAZ**), *Succinate Dehydrogenase Subunit A* (**SDHA**), 18s. The 2 most stable genes, GAPDH and HMBS, were used as reference genes to normalize the expression of jejunal target genes. The obtained data were used in statistical software for final analysis.

**Table 3:** Sequences of primer pairs used for qPCR analysis of listed references and target genes in the jejunum of necrotic enteritis challenged chickens.^1^

Genes^1^	Sequence (5′→ 3′)	Ta^°C^	Amplicon size (bp)	References
Reference genes				
*GAPDH*	F- GAAGCTTACTGGAATGGCTTTCC R: CGGCAGGTCAGGTCAACAA	61	66	Kuchipudi et al., 2012
*HMBS*	F: GGCTGGGAGAATCGCATAGG R: TCCTGCAGGGCAGATACCAT	60	131	Yin et al., 2011
Integrity genes				
*ACOX2*	F: CACTGTGCCCAGGTATAACTGC R: GACCCACGCCTTACATAGGTG	60	163	Gharib-Naseri et al., 2020
*ZO1*	F: GGATGTTTATTTGGGCGGC R: GTCACCGTGTGTTGTTCCCAT	60	187	Zanu et al., 2020
*OCLN*	F: ACGGCAGCACCTACCTCAA R: GGGCGAAGAAGCAGATGAG	60	123	Du, et al., 2016
*JAM2*	F: AGACAGGAACAGGCAGTGCTAG R: ATCCAATCCCATTTGAGGCTAC	60	135	Zanu et al., 2020
*CLDN1*	F:CTTCATCATTGCAGGTCTGTCAG R:AAATCTGGTGTTAACGGGTGTG	60	103	Zanu et al., 2020
*CLDN5*	F:GCAGGTCGCCAGAGATACAG R:CCACGAAGCCTCTCATAGCC	61	162	Zanu et al., 2020
*E-cadherins*	F: GCAAGCCGTTTACCACATCA R: GGTGGGGAGAAGGGTTGAG	61	178	Dao et al., 2022
Immunity genes				
*CHPT1*	F: CGAGCAGGCACCTTTTTGG R: GCTATGCAGGATCCAAGGACA	60	183	Gharib-Naseri et al., 2020
*IFN-γ*	F: GTGAAGAAGGTGAAAGATATCATGGA R: GCTTTGCGCTGGATTCTCA	60	71	Lammers et al., 2010
*IgA*	F: GTCACCGTCACCTGGACTACA R: ACCGATGGTCTCCTTCACATC	61	192	Lammers et al., 2010
*IgG*	F: ATCACGTCAAGGGATGCCCG R: GCATCAGCGTCACCGAAAGC	60	118	Zhao et al., 2013
*IL-12-b*	F- TGGGCAAATGATACGGTGAA R: CAGAGTAGTTCTTTGCCTCACATTTT	60	83	Shack et al., 2008
*IL-21R*	F: CTGGGAGACTCAGAAGATCAAATC R: GGTCTGGCTCTCACTTGGAATTC	60	111	Gharib-Naseri et al., 2020
*DUSP4*	F: ATCACAGCCCTGCTGAACGT R: CAGCACTCTTTCACTGAGTCGATG	60	155	Gharib-Naseri et al., 2020
*MUC2*	F: CCCTGGAAGTAGAGGTGACTG R: TGACAAGCCATTGAAGGACA	60	143	Fan et al., 2015
*NOS2*	F: CAGCTAAAGAGCCAAAAGCGA R: GTTCATGCCCGGACCAATG	60	107	Musigwa, 2020
*ZAP70*	F: GCTGGACCTACAGTTGGGAAGA R: CAATGCTGTAGTAGTAGGTGCGGA	60	110	Gharib-Naseri et al., 2020
*PEX13*	F: TGGGAGAACCGGCGATTAGT R: CAAGCCACCGTATCCATAACTG	60	264	Gharib-Naseri et al., 2020
Transporters genes				
*PepT1*	F: TACGCATACTGTCACCATCA R: TCCTGAGAACGGACTGTAAT	60	205	Guo et al., 2014
*PepT2*	F: TGACTGGGCATCGGAACAA R: ACCCGTGTCACCATTTTAACCT	60	63	Paris and Wong, 2013
*ASBT*	F: GTGGGTTATCACACCTAAGTTATG R: CACTGTACGACATCTGCTCCAAG	60	119	Gharib-Naseri et al., 2020
*APN*	F:AATACGCGCTCGAGAAAACC R:AGCGGGTACGCCGTGTT	60	70	Gilbert et al., 2007
*ACOX2*	F: CACTGTGCCCAGGTATAACTGC R: GACCCACGCCTTACATAGGTG	60	163	Gharib-Naseri et al., 2020

1 Gene names: OCLN: *occludin*, CLDN1: *claudin1*, CLDN5: *claudin5*, JAM2: *Junctional adhesion molecule2*, ZO1: *Zonula occludens-1* (tight junction protein-1), PEPT1: *peptide transporter-1*, PEPT2: *Peptide transporter-2*, ACOX2: *acyl-coa oxidase 2*, ASBT: *apical sodium-dependent bile acid transporter*, APN: *aminopeptidase N*, E-cadherins: *epithelial cadherins, Ig A: immunoglobulin A*, Ig G: *immunoglobulin G*, MUC2: *mucin 2*, IFN-γ: *interferon gamma*, IL-6: *interleukin 6*, IL-12: *interleukin 12*, IL-21R: *interleukin 21 receptor*, NOS2: *nitric oxide synthase 2*, PEX13: *peroxisomal protein*, ZAP70: *zeta chain of T cell receptor*, DUSP4: *dual specificity phosphatase 4*, CHPT1: *choline phosphotransferase 1*, HMBS: *hydroxymethylbilane synthase,* GAPDH: *β-actin, glyceraldehyde 3-phosphate dehydrogenase,*

### Statistical Analysis

All data were checked for normal distribution using JMP 14.0 (SAS Institute, USA, 2018). The experiment was arranged in a completely randomized design and the means between treatments were compared using Tukey's test, and probability values less than 0.05 were considered statistically significant. Since the distribution of lesion score data was not normal, the nonparametric test of Kruskal-Wallis was used.

## RESULTS

### Performance

Table 4 shows the effects of the NE challenge and supplemented additives on performance. At the starter phase and before inoculation of *Eimeria* (d 0–9), Balangut LS P decreased (*P* < 0.01) weight gain compared to the antibiotic group and increased (*P* < 0.01) FCR compared to nonadditive and antibiotic groups, while treatments had no significant effects on feed intake (*P* > 0.05). At the grower phase (d 9–21), NE challenge decreased (*P* < 0.001) feed intake and weight gain compared to the nonchallenged group, and additives did not affect these parameters compared to the challenged control (*P* > 0.05). FCR of all additive groups were not different (*P* > 0.05) from the challenged control, whereas the FCR of the birds fed a combination of Amasil NA and Balangut LS P did not show a difference (*P* > 0.05) from the nonchallenged group. At the finisher phase (d 21–35), all challenged birds showed no significant difference (*P* > 0.05) in feed intake and weight gain compared to the nonchallenged group. Balangut LS P and antibiotic =had lower (*P* < 0.05) FCR compared to nonchallenged control, while other challenged groups had no significant difference (*P* > 0.05) for FCR from the challenged and nonchallenged controls or antibiotic group.

**Table 4:** Effect of Amasil NA and Balangut LS P on the performance of broilers challenged with necrotic enteritis.^1^

Treatments^2^	D 0–9			D 9–21			D 21–35		
	Feed intake (g)	Weight gain (g)	FCR (g/g)	Feed intake (g)	Weight gain (g)	FCR (g/g)	Feed intake (g)	Weight gain (g)	FCR (g/g)
Nonchallenged control	248	229^ab^	1.084^b^	1123^a^	796^a^	1.412^b^	2376	1417	1.632^a^
Challenged control	250	229^ab^	1.093^b^	936^b^	620^b^	1.511^ab^	2359	1487	1.587^ab^
Ch. + Amasil NA	246	223^ab^	1.103^ab^	954^b^	606^b^	1.579^a^	2373	1487	1.596^ab^
Ch. + Balangut LS P	247	219^b^	1.127^a^	942^b^	597^b^	1.580^a^	2273	1460	1.558^b^
Ch. + Amasil NA + Balangut LS P	247	225^ab^	1.100^ab^	912^b^	622^b^	1.465^b^	2283	1448	1.578^ab^
Ch. + ZnBac	254	234^a^	1.087^b^	938^b^	595^b^	1.575^a^	2311	1487	1.554^b^
SEM	2.315	2.445	0.007	17.63	10.05	0.0245	30.41	24.41	0.0260
*P*-value									
Treatment	0.196	0.006	0.003	<0.001	<0.001	<0.001	0.065	0.263	0.013
Sex (as a covariate)	0.699	0.790	0.890	0.154	0.0077	0.325	0.0003	0.0009	0.759

a-b values within a column with different letters differ significantly (*P* < 0.05).

1 Means are obtained after sex ratio being added to the statistical model as a covariate for performance parameters.

2 Treatment descriptions were as follows: Nonchallenged control: wheat-soybean meal based diet as a basal diet; Challenged control: basal diet challenged with NE; Ch.+Amasil NA: challenged control with 0.30% Amasil NA (BASF SE, Ludwigshafen, Germany) supplemented in feed in all periods; Ch.+Balangut LS P: challenged control with 0.50%, 0.30%, 0.20% Balangut LS P (BASF SE, Ludwigshafen, Germany) supplemented in starter, grower, and finisher, respectively; Ch.+Amasil NA+Balangut LS P: challenged control with the supplementation of a combination of Amasil NA and Balangut LS P (combination of T3 and T4); Ch.+ZnBac: challenged control with antibiotic (Zn-Bacitracin, 0.05% throughout all the phases) supplemented in feed.

### Lesion Score

The results of lesion scores are shown in Table 5. For male birds, NE lesions in the jejunum of challenged groups were higher than the nonchallenged birds (*P* < 0.01), whereas there were no differences (*P* > 0.05) observed in the duodenum and ileum among groups. For female birds, NE lesions in the jejunum of all challenged groups were significantly higher (*P* < 0.01) than in nonchallenged birds. In addition, NE lesion scores in challenged birds supplemented with Amasil NA and the combination of Amasil NA with Balangut LS P showed no difference (*P* > 0.05) from both the nonchallenged and challenged controls.

**Table 5:** Effect of Amasil NA and Balangut LS P on lesion scores of broilers challenged with necrotic enteritis.

Treatments^1^	Lesion score					
	Male			Female		
	Duodenum	Jejunum	Ileum	Duodenum	Jejunum	Ileum
Nonchallenged control	0.000	0.000^b^	0.000	0.000	0.000^b^	0.000^b^
Challenged control (Ch.)	0.187	0.562^a^	0.312	0.062	0.687^a^	0.562^a^
Ch. + Amasil NA	0.062	0.687^a^	0.375	0.062	0.750^a^	0.375^a^^b^
Ch. + Balangut LS P	0.125	0.500^a^	0.187	0.250	0.625^a^	0.562^a^
Ch. + Amasil NA + Balangut LS P	0.125	0.500^a^	0.125	0.062	0.562^a^	0.375^a^^b^
Ch. + ZnBac	0.062	0.625^a^	0.187	0.062	0.562^a^	0.625^a^
SEM	0.0992	0.1555	0.1370	0.0904	0.1397	0.1456
*P*-value	0.490	0.003	0.134	0.144	0.007	0.003

a-b values within a column with different letters differ significantly (*P* < 0.05).

1 Treatment descriptions were as follows: Nonchallenged control: wheat-soybean meal based diet as a basal diet; Challenged control: basal diet challenged with NE; Ch.+Amasil NA: challenged control with 0.30% Amasil NA (BASF SE, Ludwigshafen, Germany) supplemented in feed in all periods; Ch.+Balangut LS P: challenged control with 0.50%, 0.30%, 0.20% Balangut LS P (BASF SE, Ludwigshafen, Germany) supplemented in starter, grower, and finisher, respectively; Ch.+Amasil NA+Balangut LS P: challenged control with the supplementation of a combination of Amasil NA and Balangut LS P (combination of T3 and T4); Ch.+ZnBac: challenged control with antibiotic (Zn-Bacitracin, 0.05% throughout all the phases) supplemented in feed.

### pH and FITC-d

NE challenge decreased ileal pH across all groups compared to the nonchallenged group (Table 6), while supplementing Balangut LS P and antibiotic increased (*P* < 0.001) the ileal pH compared to the challenge control group. No differences in pH value in the cecum were observed among the groups (*P* > 0.05).

**Table 6:** Effect of Amasil NA and Balangut LS P on ileal and cecal pH and FITC-d concentration in the serum of broilers challenged with necrotic enteritis.

Treatments^1^	pH		FITC-d (ng/mL serum)
	Ileal	Cecal	
Nonchallenged control	6.72^a^	6.05	0.022^b^
Challenged control (Ch.)	5.51^c^	6.12	0.092^a^^b^
Ch. + Amasil NA	5.52^c^	6.06	0.097^a^
Ch. + Balangut LS P	5.77^b^	6.00	0.097^a^
Ch. + Amasil NA + Balangut LS P	5.64^b^^c^	5.95	0.098^a^
Ch. + ZnBac	5.78^b^	6.05	0.142^a^
SEM	0.080	0.094	0.0176
*P*-value			
Treatment	<0.001	0.8676	0.001
Sex (as a covariate)	0.2124	0.1719	0.089

a-c values within a column with different letters differ significantly (*P* < 0.05).

1 Treatment descriptions were as follows: Nonchallenged control: wheat-soybean meal based diet as a basal diet; Challenged control: basal diet challenged with NE; Ch.+Amasil NA: challenged control with 0.30% Amasil NA (BASF SE, Ludwigshafen, Germany) supplemented in feed in all periods; Ch.+Balangut LS P: challenged control with 0.50%, 0.30%, 0.20% Balangut LS P (BASF SE, Ludwigshafen, Germany) supplemented in starter, grower, and finisher, respectively; Ch.+Amasil NA+Balangut LS P: challenged control with the supplementation of a combination of Amasil NA and Balangut LS P (combination of T3 and T4); Ch.+ZnBac: challenged control with antibiotic (Zn-Bacitracin, 0.05% throughout all the phases) supplemented in feed.

All NE-challenged birds received additives had higher (*P* < 0.001) concentrations of FITC-d in their serum compared to nonchallenged birds (Table 6), whereas no significant differences were observed between birds fed additives and the challenged control group (*P* > 0.05).

### Bacterial Quantification

The results of ileal bacterial quantification are shown in Table 7. NE challenge increased (*P* < 0.001) the number of *Bacillus* and total bacteria compared to nonchallenged control, whereas there were no significant differences (*P* > 0.05) between birds supplemented with additives and challenged control birds. The numbers of *Bifidobacteria, Lactobacillus*, and Enterobacteriaceae were higher (*P* < 0.05) in the challenged groups compared to the nonchallenged control, whereas the numbers of these bacteria in the Amasil NA group and the number of *Bifidobacteria* in the combination of Amasil NA and Balangut LS P being intermediate (*P* > 0.05). Challenged birds had higher (*P* < 0.001) *C. perfringens* counts in the ileum compared to nonchallenged, while the antibiotic group shifted the load of *C. perfringens* towards the nonchallenged controls (*P* > 0.05). Supplementing NE challenged birds with additives did not affect (*P* > 0.05) the number of *Bacteroids* and *Ruminococcus* in the ileum.

**Table 7:** Effect of Amasil NA and Balangut LS P on ileal microbiota (log_10_ genomic DNA copies per gram digesta) of broilers challenged with necrotic enteritis.

Treatments^1^	*Bacillus*	*Bacteroids*	*Bifidobacteria*	Enterobacteriaceae	*Lactobacillus*	*Ruminococcus*	*C. perfringens*	Total bacteria
Nonchallenged control	5.20^b^	4.87	5.60^b^	6.40^b^	7.57^b^	5.83	4.97^c^	8.45^b^
Challenged control (Ch.)	6.39^a^	4.92	6.23^a^	7.48^a^	8.58^a^	5.79	6.42^ab^	9.36^a^
Ch. + Amasil NA	6.30^a^	4.86	5.92^a^^b^	7.17^ab^	8.14^ab^	5.78	6.89^a^	9.38^a^
Ch. + Balangut LS P	6.58^a^	4.96	6.30^a^	7.42^a^	8.42^a^	6.01	7.02^a^	9.51^a^
Ch. + Amasil NA + Balangut LS P	6.25^a^	4.96	6.19^ab^	7.38^a^	8.51^a^	5.99	6.64^a^	9.36^a^
Ch. + ZnBac	6.03^a^	4.72	5.93^ab^	7.49^a^	7.93^ab^	5.70	5.22^b^^c^	9.24^a^
SEM	0.158	0.111	0.148	0.204	0.212	0.211	0.303	0.166
*P*-value								
Treatment	<0.001	0.620	0.015	0.003	0.011	0.881	<0.001	<0.001
Sex (as a covariate)	0.188	0.281	0.521	0.033	0.974	0.430	0.517	0.279

a-c Values within a column with different letters differ significantly (*P*< 0.05).

1 Treatment descriptions were as follows: Nonchallenged control: wheat-soybean meal based diet as a basal diet; Challenged control: basal diet challenged with NE; Ch.+Amasil NA: challenged control with 0.30% Amasil NA (BASF SE, Ludwigshafen, Germany) supplemented in feed in all periods; Ch.+Balangut LS P: challenged control with 0.50%, 0.30%, 0.20% Balangut LS P (BASF SE, Ludwigshafen, Germany) supplemented in starter, grower, and finisher, respectively; Ch.+Amasil NA+Balangut LS P: challenged control with the supplementation of a combination of Amasil NA and Balangut LS P (combination of T3 and T4); Ch.+ZnBac: challenged control with antibiotic (Zn-Bacitracin, 0.05% throughout all the phases) supplemented in feed.

In the cecum, NE-challenged birds supplemented with the combination of Amasil NA and Balangut LS P had a lower (*P* <0.001) number of *Bacteroids* compared to nonchallenged control (Table 8), whereas all other NE-challenged groups showed no significant differences (*P* > 0.05) with nonchallenged control. NE-challenged birds supplemented with the antibiotic had a higher (*P* < 0.05) count of Enterobacteriaceae compared to the nonchallenged control. Furthermore, the antibiotic group significantly decreased (*P* < 0.001) the number of *C. perfringens* compared to other NE-challenged groups and made a shift (*P* > 0.05) towards nonchallenged control*.* The populations of *Bacillus, Bifidobacteria, Lactobacillus, Ruminococcus*, and total bacteria in the cecum were not affected by NE challenge and additives (*P* > 0.05).

**Table 8:** Effect of Amasil NA and Balangut LS P on cecal microbiota (log_10_ genomic DNA copies per gram digesta) of broilers challenged with necrotic enteritis.

Treatments^1^	*Bacillus*	*Bacteroids*	*Bifidobacteria*	Enterobacteriaceae	*Lactobacillus*	*Ruminococcus*	*C. perfringens*	Total bacteria
Nonchallenged control	6.66	5.56^a^	7.90	8.36^b^	8.52	9.16	6.80^bc^	10.61
Challenged control (Ch.)	6.56	5.41^ab^	7.88	8.95^ab^	9.04	8.91	8.05^ab^	10.76
Ch. + Amasil NA	6.76	5.36^ab^	7.76	8.96^ab^	8.97	8.85	8.78^a^	10.72
Ch. + Balangut LS P	6.26	5.44^a^	7.74	8.96^ab^	8.88	9.01	8.06^ab^	10.72
Ch. + Amasil NA + Balangut LS P	6.70	5.21^b^	7.67	8.91^ab^	8.74	8.59	8.57^a^	10.66
Ch. + ZnBac	6.46	5.41^ab^	7.82	9.32^a^	8.81	9.00	5.55^c^	10.75
SEM	0.173	0.050	0.096	0.180	0.150	0.142	0.361	0.087
*P*-value								
Treatment	0.326	<0.001	0.473	0.019	0.202	0.126	<0.001	0.807
Sex (as a covariate)	0.966	0.738	0.748	0.089	0.041	0.817	0.596	0.091

a-c Values within a column with different letters differ significantly (*P* < 0.05).

1 Treatment descriptions were as follows: Nonchallenged control: wheat-soybean meal based diet as a basal diet; Challenged control: basal diet challenged with NE; Ch.+Amasil NA: challenged control with 0.30% Amasil NA (BASF SE, Ludwigshafen, Germany) supplemented in feed in all periods; Ch.+Balangut LS P: challenged control with 0.50%, 0.30%, 0.20% Balangut LS P (BASF SE, Ludwigshafen, Germany) supplemented in starter, grower, and finisher, respectively; Ch.+Amasil NA+Balangut LS P: challenged control with the supplementation of a combination of Amasil NA and Balangut LS P (combination of T3 and T4); Ch.+ZnBac: challenged control with antibiotic (Zn-Bacitracin, 0.05% throughout all the phases) supplemented in feed.

### mRNA Abundance

The mRNA abundance related to gut integrity and transporters is shown in Table 9. Amasil NA upregulated the mRNA abundance of ASBT compared to the nonchallenged control (*P* < 0.01), whereas other challenged groups did not affect the expression of ASBT (*P* > 0.05). Neither the NE challenge nor supplementing additives affected the abundance of gut integrity and nutrient transporters mRNA (*P* > 0.05).

**Table 9:** Effect of Amasil NA and Balangut LS P on jejunal expression of gut integrity- and transporter-related genes of male broilers challenged with necrotic enteritis.

Treatments^1^	OCLD^2^	CLDN1	CLDN5	JAM2	ZO1	PEPT1	PEPT2	ACOX2	ASBT	APN	E-cadherins
Nonchallenged control	1.219	0.907	1.173	1.316	1.215	1.389	1.633	1.143	0.043^b^	1.425	0.621
Challenged control (Ch.)	1.002	1.386	0.981	1.062	1.006	1.151	2.005	0.650	0.519^ab^	1.037	0.646
Ch. + Amasil NA	1.153	1.291	1.107	1.008	1.083	1.018	2.213	0.853	1.093^a^	1.126	0.497
Ch. + Balangut LS P	1.085	1.099	1.164	0.990	1.084	1.032	1.868	0.871	0.724^ab^	1.085	0.433
Ch. + Amasil NA + Balangut LS P	0.940	1.085	1.060	1.127	0.983	1.017	8.469	0.755	0.692^ab^	0.932	0.484
Ch. + ZnBac	0.914	0.932	1.054	0.951	1.016	0.929	1.886	0.605	0.716^ab^	0.903	0.467
SEM	0.1334	0.1820	0.1719	0.1450	0.1368	0.1542	2.9564	0.1418	0.167	0.1480	0.0835
*P*-value	0.543	0.374	0.969	0.532	0.859	0.337	0.541	0.128	0.004	0.179	0.398

a-b Values within a column with different letters differ significantly (*P* < 0.05).

1 Treatment descriptions were as follows: Nonchallenged control: wheat-soybean meal based diet as a basal diet; Challenged control: basal diet challenged with NE; Ch.+Amasil NA: challenged control with 0.30% Amasil NA (BASF SE, Ludwigshafen, Germany) supplemented in feed in all periods; Ch.+Balangut LS P: challenged control with 0.50%, 0.30%, 0.20% Balangut LS P (BASF SE, Ludwigshafen, Germany) supplemented in starter, grower, and finisher, respectively; Ch.+Amasil NA+Balangut LS P: challenged control with the supplementation of a combination of Amasil NA and Balangut LS P (combination of T3 and T4); Ch.+ZnBac: challenged control with antibiotic (Zn-Bacitracin, 0.05% throughout all the phases) supplemented in feed.

2 OCLD: occludin, CLDN1: claudin1, CLDN5: claudin5, JAM2: Junctional adhesion molecule2, ZO1: Zonula occludens-1 (tight junction protein-1), PEPT1: peptide transporter-1, PEPT2: Peptide transporter-2, ACOX2: acyl-coa oxidase 2, ASBT: apical sodium-dependent bile acid transporter, APN: aminopeptidase N, E-cadherins: epithelial cadherins.

Table 10 shows the effects of NE challenge and additives on the abundance of immune-related mRNA in the jejunum of chickens on d 16. NE challenge upregulated the abundance of IFN-γ (*P* < 0.001), IL-21R (*P* < 0.001), ZAP70 (*P* < 0.01), and DUSP4 (*P* < 0.01) compared to nonchallenged birds and also moved the expression of PEX13 towards the nonchallenged group (*P* > 0.05). All additives upregulated (*P* < 0.001) the expression of IFN-γ in the jejunum of challenged birds compared to nonchallenged ones. Amasil NA and the combination of Amasil NA and Balangut LS P upregulated the mRNA abundance of IL-21R (*P* < 0.001), ZAP70 (*P* < 0.01), and DUSP4 (*P* < 0.01) compared to the nonchallenged group, whereas the expression of these genes in Balangut LS P and antibiotic groups being intermediate (*P* > 0.05). NE challenge and additives did not affect the expression of Ig A, Ig G, IL-12-b, NOS2, and CHPT1 (*P* > 0.05).

**Table 10:** Effect of Amasil NA and Balangut LS P on jejunal expression of gut immunity-related genes of male broilers challenged with necrotic enteritis.

Treatments^1^	Ig A^2^	Ig G	MUC2	IFN-γ	IL-12-b	IL-21R	NOS2	PEX13	ZAP70	DUSP4	CHPT1
Nonchallenged control	0.677	1.089	0.962	0.302^b^	0.146	0.481^b^	0.916	1.206^a^	0.349^b^	0.504^b^	0.276
Challenged control (Ch.)	0.730	1.048	1.296	1.655^a^	0.281	1.640^a^	1.154	0.747^ab^	0.948^a^	1.388^a^	0.492
Ch. + Amasil NA	0.641	1.398	1.060	1.358^a^	0.255	1.195^a^	1.259	0.570^b^	1.085^a^	1.358^a^	0.472
Ch. + Balangut LS P	0.534	0.576	1.013	1.228^a^	0.169	1.145^a^^b^	1.103	0.660^b^	0.808^a^^b^	1.220^a^^b^	0.376
Ch. + Amasil NA + Balangut LS P	0.433	1.500	1.112	1.520^a^	0.192	1.289^a^	1.040	0.564^b^	0.904^a^	1.243^a^	0.470
Ch. + ZnBac	0.524	1.130	0.911	1.343^a^	0.240	1.096^a^^b^	1.232	0.505^b^	0.852^a^^b^	1.155^a^^b^	0.458
SEM	0.1714	0.3703	0.1312	0.2092	0.0428	0.1611	0.1984	0.1138	0.1242	0.1697	0.078
*P*-value	0.847	0.582	0.391	<0.001	0.174	<0.001	0.837	0.001	0.005	0.007	0.368

a-b Values within a column with different letters differ significantly (*P* < 0.05).

1 Treatment descriptions were as follows: Nonchallenged control: wheat-soybean meal based diet as a basal diet; Challenged control: basal diet challenged with NE; Ch.+Amasil NA: challenged control with 0.30% Amasil NA (BASF SE, Ludwigshafen, Germany) supplemented in feed in all periods; Ch.+Balangut LS P: challenged control with 0.50%, 0.30%, 0.20% Balangut LS P (BASF SE, Ludwigshafen, Germany) supplemented in starter, grower, and finisher, respectively; Ch.+Amasil NA+Balangut LS P: challenged control with the supplementation of a combination of Amasil NA and Balangut LS P (combination of T3 and T4); Ch.+ZnBac: challenged control with antibiotic (Zn-Bacitracin, 0.05% throughout all the phases) supplemented in feed.

2 Ig A: immunoglobulin A, Ig G: immunoglobulin G, MUC2: mucin 2, IFN-γ: interferon gamma, IL-6: interleukin 6, IL-12: interleukin 12, IL-21R: interleukin 21 receptor, NOS2: nitric oxide synthase 2, PEX13: peroxisomal protein, ZAP70: zeta chain of T cell receptor, DUSP4: dual specificity phosphatase 4, CHPT1: choline phosphotransferase 1.

## DISCUSSION

The current study investigated the effects of a buffered formic acid (Amasil NA), a monoglycerides mixture (Balangut LS P), and their combination on NE challenged birds. The findings from this study showed that Balangut LS P improved FCR in the finisher phase and shifted the expression levels of some immune-related genes closer to the levels to the nonchallenged group. Further, Amasil NA shifted the counts of ileal bacteria towards levels closer to nonchallenged birds. The combination of Amasil NA with Balangut LS P improved FCR to a level with no difference from the nonchallenged birds at the grower phase and reduced the level of *Bacteriodes* in the cecum. Therefore, we accept the hypothesis that Balangut LS P and Amasil NA can modulate the gut microenvironment in challenged birds by supporting intestinal rehabilitation from damaged epithelial cells by the NE challenge through a different mechanism(s).

The addition of Balangut LS P to the diet of NE-challenged broilers showed greater recovery of the feed efficiency during the finisher phase (d 21–35) and shifted the expression of IL-21R, ZAP70, and DUSP4 towards the nonchallenged group. Balangut LS P is a blend of short (butyric and propionic) and medium (lauric, caprylic, and capric) chain fatty acids, which are glycerinated to avoid its degradation in the upper section of the gut so as to reach the optimum absorption site in the lower intestine, where these acids could play their physiological roles (Pituch et al., 2013; Bedford and Gong, 2018). Furthermore, intestinal homeostasis plays a crucial role in the health and growth of the host, while invasive pathogens can disrupt the intestinal mucosal layer, stimulate the immune system, and change the energy flow to immunity rather than maintenance and growth (Doeschl-Wilson et al., 2009; van der Most et al., 2011; Daneshmand et al., 2022). The beneficial effects of monoglyceride and fatty acid content of Balangut LS P on controlling *E. coli* and *C. perfringens* have been reported previously (Skřivanová et al., 2014; Gharib-Naseri et al., 2021b). In addition, Gharib-Naseri et al. (2021a) evaluated the effects of NE challenge on several genes related to the intestinal immunity of broilers. It was reported that NE infection upregulated the expression of immune-related genes related to T-cells activation, which agrees with the current results. Previous studies showed that the upregulation of immunity genes such as IL-21R, ZAP70, and DUSP4 could activate the immunological cascades secreting T cell agents such as inducible nitric oxide (Liew et al., 1990) and signal transducer and activator of transcription-3 (Spolski and Leonard, 2008), resulting in antimicrobial activity against invading pathogens. Although the exact mechanism of Balangut LS P on lowering FCR has yet to be clear, 2 possible mechanisms can be possibly attributed to the immunomodulatory effects of Balangut LS P components. Since Balangut LS P contains various amounts of monoglyceride derivatives of SMCFA, they might modulate the inflammatory responses in NE-challenged birds through the interaction of SMCFA with the G-protein-coupled receptor, as explained previously (Briscoe et al., 2003; Li et al., 2018) and supported the immune responses. The second possible mechanism has been attributed to Monobutyrin, one of the main monoglycerides in Balangut LS P. Its beneficial effects on the growth and health of chickens under bacterial challenge have been reported (Antongiovanni et al., 2011). Monobutyrin releases butyrate into the intestinal lumen (Moquet et al., 2016), showing immunostimulatory effects on broilers under normal and challenged conditions (Ao et al., 2012). Therefore, it could be assumed that Balangut LS P triggered the immune system by shifting the expression of immune-related genes of IL-21R, ZAP70, and DUSP4 to respond to pathogenic infection, possibly recovered the intestinal environment during the recovery phase and inclined energy sources from immunity to the growth, as evidenced by a lower FCR in this treatment at finisher phase. Although the mRNA abundance of the immune genes and performance results of Balangut LS P supported each other, other results of this group, such as lesion score, gut integrity, and microbiota, were not significantly different indicating an mild NE as a subclinical challenge.

Compared to a higher level of bacteria in the ileum of NE-challenged broilers, Amasil NA shifted the counts of ileal *Bifidobacteria*, Enterobacteriaceae, and *Lactobacillus* towards the nonchallenged group, whereas this additive did not significantly affect other evaluated parameters. The commensal bacteria of the intestine play critical roles in the health and growth of the host through different modes of action, including changes in intestinal pH, mucosal immunity, and nutrient absorption (Apajalahti et al., 2004). Amasil NA is mainly composed of formic acid (61% formic acid and 20.5% sodium formate), and the beneficial effects of formic acid on gut health and the immune system has been reported (Ricke et al., 2020). The results of previous experiments demonstrated the bactericidal effects of formic acid-based commercial organic acids on various pathogenic bacteria such as *S. enteritidis* (Thompson and Hinton, 1997), *S. typhimurium* (Byrd et al., 2001), and *E. coli* (Khodambashi Emami et al., 2017). The lower counts of *Bifidobacteria*, Enterobacteriaceae, and *Lactobacillus* observed in formic acid-supplemented diets may be attributed to the effects of organic acids on the alteration of short-chain fatty acids profile in the ileum (Goodarzi-Boroojeni et al., 2014; Gharib-Naseri et al., 2021b), and also the role of other organs like the crop in controlling metabolites reached to the ileum (Thompson and Hinton, 1997). However, this study showed that the antibacterial effects of formic acid in Amasil NA did not lead to the improvement of other parameters such as performance, lesion score, and gut integrity in NE-challenged broilers. This may warrant further study on the dosage and time of the Amasil application to the birds.

The combination of Amasil NA and Balangut LS P significantly reduced FCR in the grower phase and lowered the level of *Bacteroides* in cecal pouches. *Bacteroides* are common inhabitants of the chicken cecum (Yang et al., 2018), but they are opportunistic species and can become pathogenic under infectious challenges (Wexler, 2007). Furthermore, the proliferation of *Bacteroides* under disease conditions can overstimulate the immune system and impose excessive energy costs on the host (Wexler, 2007); hence, inhibiting *Bacteroides* proliferation may improve the intestinal health of birds under challenging conditions. In the current study, the combination of Amasil NA and Balangut LS P decreased the level of *Bacteroids* in the cecum of NE challenged birds, which may be attributed to the synergistic bactericidal effects of the organic acids content of these additives, as has been reported previously (Thompson and Hinton, 1997). Although the combination of additives reduced the level of *Bacteroids* in the cecum, this might not be a strong evidence to imply that this solely phenomena resulted in a lower FCR in the grower phase, while this lowering effect did not observe at the end of the experiment and also other parameters did not support the performance result. Gharib-Naseri et al. (2021b) concluded that Balangut LS P and Amasil NA could change the pH value and consequently modulate the bacterial population in the cecum of NE challenged chickens. Overall, although the combination of Amasil NA with Balangut LS P reduced the level of *Bacteroides* population in the cecum of NE-challenged birds which may led to lower FCR in the grower phase, this reduction did not mitigate the negative effects of NE on other parameters such as performance, lesion score, gut integrity, and mRNA abundance in challenged birds.

Overall, the current study showed that Balangut LS P improved FCR at the end of the experiment and shifted the expression of intestinal immune-related genes towards the nonchallenged group. Amasil NA altered the counts of ileal bacteria to the intermediate level between challenged and nonchallenged birds. The combination of both additives improved FCR at the grower phase (peak of NE challenge) and reduced the number of bacteria in the cecum of challenged birds. Since Balangut LS P, Amasil NA, and their combination improved the performance of NE challenged birds, possibly through modulatory effects on the intestinal microbiota and immune system, the hypothesis of this study is accepted. Further studies are warranted to optimize the individual levels of Balangut LS P and Amasil NA for their most effective doses individually or in combination for the improvement of the performance and health of broilers under challenging conditions.

## DISCLOSURES

The authors declared that there are no conflicts of interest.

## References

[bib0001] Aarestrup F.M., Wegener H.C., Collignon P. (2008). Resistance in bacteria of the food chain: epidemiology and control strategies. Expert. Rev. Anti. Infec. Ther..

[bib0002] Antongiovanni M., Pmassi P., Mparini M., Buccioni A., Btempesta B., Minieri S.M. (2011). Page 188 in La Monobutyrine: Un Promoteur de Croissance et Produit de Prevention et Thèrapie Contre les Infections Entèriques Comunes des Poulets de Chair. 29–30 March.

[bib0003] Ao Z., Kocher A., Choct M. (2012). Effects of dietary additives and early feeding on performance, gut development and immune status of broiler chickens challenged with *Clostridium perfringens*. Asian-Australas. J. Anim. Sci..

[bib0004] Apajalahti J., Kettunen A., Graham H. (2004). Characteristics of the gastrointestinal microbial communities, with special reference to the chicken. World Poult. Sci. J..

[bib0005] Asaoka Y., Yannai T., Hirayama H., Une Y., Saito E., Sakai H., Goryo M., Fukushi H., Masegi T. (2004). Fatal necrotic enteritis associated with *Clostridium perfringens* in wild crows (*Corvus macrorhynchos*). Avian Pathol..

[bib0006] Bartosch S., Fite A., Macfarlane G.T., McMurdo M.E. (2004). Characterization of bacterial communities in feces from healthy elderly volunteers and hospitalized elderly patients by using real-time PCR and effects of antibiotic treatment on the fecal microbiota. Appl. Environ. Microbiol..

[bib0007] Bedford A., Gong J. (2018). Implications of butyrate and its derivatives for gut health and animal production. Anim. Nutr..

[bib0008] Briscoe C.P., Tadayyon M., Andrews J.L., Benson W.G., Chambers J.K., Eilert M.M., Ellis C., Elshourbagy N.A., Goetz A.S., Minnick D.T., Murdock P.R., Sauls H.R., Shabon U., Spinage L.D., Strum J.C., Szekeres P.G., Tan K.B., Way J.M., Ignar D.M., Wilson S., Muir A.I. (2003). The orphan G protein-coupled receptor GPR40 is activated by medium and long chain fatty acids. J. Biol. Chem..

[bib0009] Byrd J.A., Hargis B.M., Caldwell D.J., Bailey R.H., Herron K.L., McReynolds J.L. (2001). Effect of lactic acid administration in the drinking water during pre-slaughter feed withdrawal on *Salmonella* and *Campylobacter* contamination of broilers. Poult. Sci..

[bib0011] Cobb-Vantress, Broiler Management guides. 2018a. Accessed August 2023. https://www.cobb-vantress.com/assets/5c7576a214/Broiler-guide-R1.pdf

[bib0010] Cobb-Vantress, Broiler nutrient recommendations. 2018b. Accessed August 2023. https://www.cobb-vantress.com/assets/5a88f2e793/Broiler-Performance-Nutrition-Supplement.pdf

[bib0012] Daneshmand A., Kermanshahi H., Mohammed J., Sekhavati M.H., Javadmanesh A., Ahmadian M., Alizadeh M., Razmyar J., Kulkarni R.R. (2022). Intestinal changes and immune responses during *Clostridium perfringens*-induced necrotic enteritis in broiler chickens. Poult. Sci..

[bib0013] Dao H.T., Sharma N.K., Kheravii S.K., Bradbury E.J., Wu S.-B., Swick R.A. (2022). Supplementation of reduced protein diets with L-arginine and L-citrulline for broilers challenged with subclinical necrotic enteritis. 3. Immunological parameters and gene expression. Anim. Prod. Sci..

[bib0014] Doeschl-Wilson A.B., Brindle W., Emmans G., Kyriazakis I. (2009). Unravelling the relationship between animal growth and immune response during micro-parasitic infections. PLoS One.

[bib0015] Du E., Wang W., Gan L., Li Z., Guo S., Guo Y. (2016). Effects of thymol and carvacrol supplementation on intestinal integrity and immune responses of broiler chickens challenged with *Clostridium perfringens*. J. Anim. Sci. Biotechnol..

[bib0016] England A.D., Kheravii S.K., Musigwa S., Kumar A., Daneshmand A., Sharma N.K., Gharib-Naseri K., Wu S.-B. (2021). Sexing chickens (*Gallus gallus domesticus*) with high-resolution melting analysis using feather crude DNA. Poult. Sci..

[bib0017] Fan X., Liu S., Liu G., Zhao J., Jiao H., Wang X., Song Z., Lin H. (2015). Vitamin A deficiency impairs mucin expression and suppresses the mucosal immune function of the respiratory tract in chicks. PLoS One.

[bib0018] Fu C.J., Carter J.N., Li Y., Porter J.H., Kerley M.S. (2006). Comparison of agar plate and real-time PCR on enumeration of Lactobacillus, *Clostridium perfringens* and total anaerobic bacteria in dog faeces. Lett. Appl. Microbiol..

[bib0021] Gharib-Naseri K., de Las Heras-Saldana S., Kheravii S.K., Qin Qin L., Wang J., Wu S.-B. (2021). Necrotic enteritis challenge effects PPAR signaling and β-oxidation pathways in broiler chicken transcriptome. Anim. Nutr..

[bib0019] Gharib-Naseri K., de Paula Dorigam J.C., Doranalli K., Kheravii S.K., Swick R.A., Choct M., Wu S.-B. (2020). Modulations of genes related to gut integrity, apoptosis, and immunity underlie the beneficial effects of Bacillus amyloliquefaciens CECT 5940 in broilers fed diets with different protein levels in a necrotic enteritis challenge model. J. Anim. Sci. Biotechnol..

[bib0020] Gharib-Naseri K., Kheravii S.K., Li L., Wu S.B. (2021). Buffered formic acid and a monoglyceride blend coordinately alleviate subclinical necrotic enteritis impact in broiler chickens. Poult. Sci..

[bib0022] Gilbert E., Li H., Emmerson D., Webb K., Wong E. (2007). Developmental regulation of nutrient transporter and enzyme mRNA abundance in the small intestine of broilers. Poult. Sci..

[bib0023] Goodarzi-Boroojeni F., Vahjen W., Mader A., Knorr F., Ruhnke I., Röhe I. (2014). The effects of different thermal treatments and organic acid levels in feed on microbial composition and activity in gastrointestinal tract of broilers. Poult. Sci..

[bib0024] Guo S., Liu D., Zhao X., Li C., Guo Y. (2014). Xylanase supplementation of a wheat-based diet improved nutrient digestion and mRNA expression of intestinal nutrient transporters in broiler chickens infected with *Clostridium perfringens*. Poult. Sci..

[bib0025] Hajati H. (2018). Application of organic acids in poultry nutrition. Int. J. Avian. Wildlife Biol..

[bib0026] Han G.Q., Xiang Z.T., Yu B., Chen D.W., Qi H.W., Mao X.B., Chen H., Mao Q., Huang Z.Q. (2012). Effects of different starch sources on Bacillus spp. in intestinal tract and expression of intestinal development related genes of weanling piglets. Mol. Biol. Rep..

[bib0027] Hernández F., García V., Madrid J., Orengo J., Catalá P., Megías M.D. (2006). Effect of formic acid on performance, digestibility, intestinal histomorphology and plasma metabolite levels of broiler chickens. Br. Poult. Sci..

[bib0028] Keyburn A.L., Sheedy S.A., Ford M.E., Williamson M.M., Awad M.M., Rood J.I., Moore R.J. (2006). Alpha-toxin of *Clostridium perfringens* is not an essential virulence factor in necrotic enteritis in chickens. Infect. Immun..

[bib0029] Kheravii S.K., Swick R.A., Choct M., Wu S.B. (2018). Effect of oat hulls as a free choice feeding on broiler performance, short chain fatty acids and microflora under a mild necrotic enteritis challenge. Anim. Nutr..

[bib0030] Khodambashi Emami N., Daneshmand A., Naeini S.Z., Graystone E.N., Broom L.J. (2017). Effects of commercial organic acid blends on male broilers challenged with *E. coli* K88: Performance, microbiology, intestinal morphology, and immune response. Poult. Sci..

[bib0031] Kuchipudi S.V., Tellabati M., Nelli R.K., White G.A., Perez B.B., Sebastian S., Slomka M.J., Brookes S.M., Brown I.H., Dunham S.P. (2012). 18S rRNA is a reliable normalisation gene for real time PCR based on influenza virus infected cells. Virol. J..

[bib0032] Kumar A., Toghyani M., Kheravii S.K., Pineda L., Han Y., Swick R.A., Wu S.B. (2021). Potential of blended organic acids to improve performance and health of broilers infected with necrotic enteritis. Anim. Nutr..

[bib0033] Lammers A., Wieland W.H., Kruijt L., Jansma A., Straetemans T., Schots A., Hartog G., Parmentier H.K. (2010). Successive immunoglobulin and cytokine expression in the small intestine of juvenile chicken. Dev. Comp. Immunol..

[bib0034] Layton A., McKay L., Williams D., Garrett V., Gentry R., Sayler G. (2006). Development of bacteroides 16S rRNA gene TaqMan-based real-time PCR assays for estimation of total, human, and bovine fecal pollution in water. Appl. Environ. Microbiol..

[bib0035] Lee D.-H., Zo Y.-G., Kim S.-J. (1996). Nonradioactive method to study genetic profiles of natural bacterial communities by PCR single-strand-conformation polymorphism. Appl. Environ. Microbiol..

[bib0036] Li M., van Esch B.C.A.M., Wagenaar G.T.M., Garssen J., Folkerts G., Henricks P.A.J. (2018). Pro- and anti-inflammatory effects of short chain fatty acids on immune and endothelial cells. Eur. J. Pharmacol..

[bib0037] Liew F., Millott S., Parkinson C., Palmer R., Moncada S. (1990). Macrophage killing of Leishmania parasite in vivo is mediated by nitric oxide from L-arginine. J. Immunol..

[bib0039] Moquet P.C.A., Onrust L., Van Immerseel F., Ducatelle R., Hendriks W.H., Kwakkel R.P. (2016). Importance of release location on the mode of action of butyrate derivatives in the avian gastrointestinal tract. World Poult. Sci. J..

[bib0040] Musigwa S. (2020).

[bib0041] Paris N., Wong E. (2013). Expression of digestive enzymes and nutrient transporters in the intestine of Eimeria maxima-infected chickens. Poult. Sci..

[bib0042] Pituch A., Walkowiak J., Banaszkiewicz A. (2013). Butyric acid in functional constipation. Prz Gastroenterol..

[bib0043] Ragaa N.M., Korany R. (2016). Studying the effect of formic acid and potassium diformate on performance, immunity and gut health of broiler chickens. Anim. Nutr..

[bib0044] Ramirez-Farias C., Slezak K., Fuller Z., Duncan A., Holtrop G., Louis P. (2008). Effect of inulin on the human gut microbiota: stimulation of *Bifidobacterium adolescentis* and *Faecalibacterium prausnitzii*. Br. J. Nutr..

[bib0045] Requena T., Burton J., Matsuki T., Munro K., Simon M.A., Tanaka R., Watanabe K., Tannock G.W. (2002). Identification, detection, and enumeration of human Bifidobacterium species by PCR targeting the transaldolase gene. Appl. Environ. Microbiol..

[bib0047] Ricke S.C., Dittoe D.K., Richardson K.E. (2020). Formic acid as an antimicrobial for poultry production: a review. Front. Vet. Sci..

[bib0048] Rodgers N.J., Swick R.A., Geier M.S., Moore R.J., Choct M., Wu S.–B. (2015). A multifactorial analysis of the extent to which Eimeria and fishmeal predispose broiler chickens to necrotic enteritis. Avian Dis..

[bib0049] SAS. Institute (2018).

[bib0050] Shack L.A., Buza J.J., Burgess S.C. (2008). The neoplastically transformed (CD30hi) Marek's disease lymphoma cell phenotype most closely resembles T-regulatory cells. Can. Immunol. Immunother..

[bib0051] Skřivanová E., Pražáková Š., Benada O., Hovorkova P., Marounek M. (2014). Susceptibility of *Escherichia coli* and *Clostridium perfringens* to sucrose monoesters of capric and lauric acid. Czech J. Anim. Sci..

[bib0052] Spolski R., Leonard W.J. (2008). The Yin and Yang of interleukin-21 in allergy, autoimmunity and cancer. Curr. Opin. Immunol..

[bib0053] Thompson J.L., Hinton M. (1997). Antibacterial activity of formic and propionic acids in the diet of hens on salmonellas in the crop. Br. Poult. Sci..

[bib0056] van der Most P.J., de Jong B., Parmentier H.K., Verhulst S. (2011). Trade-off between growth and immune function: a meta-analysis of selection experiments. Funct. Ecol..

[bib0057] Van Immerseel F., Russell J.B., Flythe M.D., Gantois I., Timbermont L., Pasmans F., Haesebrouck F., Ducatelle R. (2006). The use of organic acids to combat Salmonella in poultry: a mechanistic explanation of the efficacy. Avian Pathol..

[bib0058] Vicuña E.A., Kuttappan V.A., Galarza-Seeber R., Latorre J.D., Faulkner O.B., Hargis B.M., Tellez G., Bielke L.R. (2015). Effect of dexamethasone in feed on intestinal permeability, differential white blood cell counts, and immune organs in broiler chicks. Poult. Sci..

[bib0059] Wade B., Keyburn A.L. (2015). World Poult..

[bib0061] Wexler H.M. (2007). Bacteroides: the good, the bad, and the nitty-gritty. Clin. Microbiol. Rev..

[bib0062] Wise M.G., Siragusa G.R. (2005). Quantitative detection of *Clostridium perfringens* in the broiler fowl gastrointestinal tract by real-time PCR. Appl. Environ. Microbiol..

[bib0064] Yang X., Yin F., Yang Y. (2018). Dietary butyrate glycerides modulate intestinal microbiota composition and serum metabolites in broilers. Sci. Rep..

[bib0065] Yin R., Liu X., Liu C., Ding Z., Zhang X., Tian F., Liu W., Yu J., Li L., de Angelis M.H. (2011). Systematic selection of housekeeping genes for gene expression normalization in chicken embryo fibroblasts infected with Newcastle disease virus. Biochem. Biophys. Res. Commun..

[bib0066] Zanu H.K., Kheravii S.K., Morgan N.K., Bedford M.R., Swick R.A. (2020). Interactive effect of dietary calcium and phytase on broilers challenged with subclinical necrotic enteritis: part 2. Gut permeability, phytate ester concentrations, jejunal gene expression, and intestinal morphology. Poult. Sci..

[bib0067] Zhao F.Q., Zhang Z.W., Yao H.D., Wang L.L., Liu T., Yu X.Y., Li S., Xu S.W. (2013). Effects of cold stress on mRNA expression of immunoglobulin and cytokine in the small intestine of broilers. Res. Vet. Sci..

